# Maternal Stress, Depression, and Attachment in the Neonatal Intensive Care Unit Before and During the COVID Pandemic: An Exploratory Study

**DOI:** 10.3389/fpsyg.2021.734640

**Published:** 2021-10-01

**Authors:** Filippa Manuela, Francisca Barcos-Munoz, Maria Grazia Monaci, Lara Lordier, Maricé Pereira Camejo, Joana Sa De Almeida, Didier Grandjean, Petra S. Hüppi, Cristina Borradori-Tolsa

**Affiliations:** ^1^Division of Development and Growth, Department of Pediatrics, Obstetrics and Gynaecology, University of Geneva, Geneva, Switzerland; ^2^Neuroscience of Emotion and Affective Dynamics Lab, Swiss Center for Affective Sciences, Faculty of Psychology and Educational Sciences, University of Geneva, Geneva, Switzerland; ^3^Department of Human and Social Sciences, University of Valle d’Aosta, Aosta, Italy; ^4^Division of Pediatric Intensive Care and Neonatology, Department of Women, Children and Adolescents, University Hospital of Geneva, Geneva, Switzerland

**Keywords:** neonatal intensive care unit, preterm infants, maternal stress, maternal depression, attachment, COVID pandemic

## Abstract

The main aim of the present study was to investigate the effects of the COVID-19 pandemic on the mothers’ postnatal depression, stress, and attachment during their stay in the Neonatal Intensive Care Unit (NICU). Twenty mothers of very premature infants born before 32weeks of gestational age were recruited at the Geneva University Hospital between January 2018 and February 2020 before the COVID-19 pandemic started. Mothers were screened for postnatal depression after their preterm infant’s birth (Edinburgh Postnatal Depression Scale, EPDS), then for stress (Parental Stressor Scale: Neonatal Intensive Care Unit, PSS:NICU), and attachment (Maternal Postnatal Attachment Scale, MPAS) at infant’s term-equivalent age. Data were compared with 14 mothers recruited between November 2020 and June 2021 during the COVID-19 pandemic. No significant differences were found in the scores for depression, stress, and attachment between the two groups. However, a non-statistically significant trend showed a general increase of depression symptoms in mothers during the COVID-19 pandemic, which significantly correlated to the attachment and stress scores. Moreover, the PSS:NICU Sights and Sounds score was significantly positively correlated with EPDS scores and negatively with the MPAS score only in the During-COVID group. To conclude, we discussed a possible dampened effect of the several protective family-based actions that have been adopted in the Geneva University Hospital during the health crisis, and we discussed the most appropriate interventions to support parents in this traumatic period during the COVID-19 pandemic.

## Introduction

The COVID-19 pandemic has been shown to have important public health implications including emotional and social functioning, particularly for populations at risk (Pfefferbaum and North, 2020). It is therefore becoming a priority to investigate its effects on vulnerable populations of newborns. Women who gave birth during the new COVID-19 pandemic showed an increased risk of traumatic birth experiences compared to women who gave birth before the pandemic period (Mayopoulos et al., 2021). Moreover, higher levels of depression and symptoms of anxiety have been documented in the postnatal period since the beginning of the COVID-19 pandemic (Cameron et al., 2020; Davenport et al., 2020; Davis-Floyd et al., 2020; Ahmad and Vismara, 2021). During their child’s hospitalization in the neonatal intensive care unit (NICU), mothers of premature babies face not only the known stressors of premature birth but also the issues related to the pandemic (Tscherning et al., 2020).

In the NICUs, although the World Health Organization has recommended healthcare providers to promote close contact, skin-to-skin, and rooming-in throughout the day and night including, during COVID-19 pandemic (WHO and EMRO), the vast majority of NICUs needed to adopt major changes in their care during the emergency period (van Veenendaal et al., 2021). The evident consequences of isolation measures included an increased risk of separation, stress, and depression for parents of preterm infants hospitalized in the NICUs. During periods of restricted visits to their hospitalized baby due to the COVID-19 pandemic, parents expressed psychological distress that could have detrimental effects on the parent–child relationship (van Veenendaal et al., 2021). In several NICUs, the parental access was restricted to one parent only – usually the mother – and the fathers could meet their newborn preterm infants only after hospital discharge (Ancora and Simone, 2021). Moreover, the time that a parent could spend with their infant was significantly reduced and sometimes limited to few hours per day (Lavizzari et al., 2021). In extreme cases, the parental presence was completely denied, and parents could not visit their infants (Muniraman et al., 2020). Parents reported significant impacts on their ability to visit, care for, and bond with their child, and this negative perception was more evident in hospitals with stricter restrictions (Bembich et al., 2020; Muniraman et al., 2020). During periods of restricted visits to their hospitalized baby due to the COVID-19 pandemic, parents expressed psychological distress that could have detrimental effects on the parent–child relationship (van Veenendaal et al., 2021). It is known that the separation policies in the NICU, during sensitive periods of infants’ brain development, have detrimental effects on infants (Flacking et al., 2012) and on mother-infant bonding experience (Korja et al., 2012). During the pandemic, NICU policies preserving 24-h parental presence decreased significantly (Mahoney et al., 2020), resulting in a reduction in therapeutic services (i.e., physiotherapy, music therapy, etc.), lactation interventions, and, more generally, in parenting support.

In addition, not only parents but also the staff organization was impacted by the COVID-19 pandemic with consequences on infant’s care: the staff was redeployed and non-urgent procedures, such as multidisciplinary therapies, were delayed (Mahoney et al., 2020).

However, no studies at our knowledge investigated the impact of the COVID pandemic on the maternal depression, stress, and attachment scores, by comparing the two periods, before and during the COVID pandemic.

Following the overmentioned, the main objective of the present study was to investigate the effects of the COVID-19 pandemic on the mothers’ depression and stress, as well as on the attachment process with their child during the NICU stay. We hypothesized that the COVID-19 pandemic could have a detrimental impact on maternal depression after birth, on maternal stress symptoms during their hospital stay in the NICU, and, consequently, on maternal attachment at hospital discharge.

## Materials and Methods

### Participants and Procedure

Participants were recruited at the University Hospital of Geneva before and during the COVID-19 pandemic. Thirty-four mothers were involved in the study: 20 in the Before-COVID period and 14 in the During-COVID period.

COVID-19 was declared a global pandemic by the World Health Organization (WHO) on March 11, 2020 (Cucinotta and Vanelli, 2020). Mothers of infants born very prematurely, before 32weeks of gestation, in the Before-COVID group were recruited between January 2018 and February 2020, while mothers of infants born very prematurely in the During-COVID group were recruited between November 2020 and June 2021. Women with overt psychiatric symptoms and needing specific psychological or psychiatric treatment were excluded.

Participants were individually approached by the medical staff of the NICU. The Swiss Ethical Committee approved the study and written informed consents were obtained.

Clinical neonatal characteristics of the infants of the recruited mothers in both groups were routinely recorded. Gestational age was based on the best estimate from early ultrasound or last menstrual period. Neonatal sepsis, bronchopulmonary dysplasia (PBD), and major brain injury (such as intraventricular haemorrhage grade 3 or 4 and/or cystic leukomalacia) were defined as previously published (Schlapbach et al., 2012).

Mothers’ and infants’ characteristics are reported in Table 1 and were collected in the hospital digital clinical records. To complete the population description, mothers were asked to complete a questionnaire on their Socioeconomic status (SES). The SES was estimated using a validated score based on maternal education and paternal occupation (score ranging from 2 to 12). A lower SES score reflects a higher socio-economic level (Largo et al., 1989).

**Table 1:** Infants’ and mothers’ characteristics in the Before- and During-COVID groups.

	Before-COVID Mean (SD; *N*=20)	During-COVID Mean (SD; *N*=14)	*p*
**Preterm infants**			
Gestational age (weeks)	29.45 (6.8)	29.03 (13.5)	0.57
Birth weight (g)	1257.25 (388)	1232.5 (437.4)	0.85
Height at birth (cm)	38.63 (3.0)	37.8 (14.98)	0.5
Head circumference at birth (cm)	26.94 (2.4)	26.11 (3.2)	0.37
Bronchopulmonary dysplasia (yes/total)	6 (30%)	9 (64%)	0.2
Proven sepsis (yes/total)	3 (15%)	5 (35%)	0.33
Intraventricular hemorrhage grade 3–4 (yes/total)	0	0	
Periventricular leukomalacia (yes/total)	0	0	
**Mothers**			
Age (<29; 30–39; >40)	9 (45%); 9 (45%); 2 (10%)	3 (21%); 6 (43%); 5 (36%)	0.06
Type of pregnancy (singleton/total, %)	14/20 (70,0%)	10/14 (71,4%)	0.6
Type of delivery (cesarean/total, %)	15/20 (75,0%)	10/14 (71,4%)	0.56
Primiparous (yes/total)	11/20 (55,0%)	11/14 (78%)	0.15
Marital status (couple/total)	18/20 (90,0%)	13/14 (92,9%)	0.64

## Measures

A first questionnaire was administered when infants reached 33weeks of gestational age – the Edinburgh Postnatal Depression Scale (EPDS) – and two others when the infant reached term-equivalent age, the Parental Stressor Scale: Neonatal Intensive Care Unit (PSS:NICU) and the Maternal Postnatal Attachment Scale (MPAS).

The EPDS was developed to assist health professionals in detecting mothers suffering from postpartum depression, a more prolonged disorder than the “blues” (which can occur in the 1st week after delivery). The scale consists of 10 short statements. A mother checks off one of four possible answers that are the closest to how she has felt during the past week. Responses are scored 0, 1, 2, and 3 based on the seriousness of the symptom (minimum total score=0 and maximum total score=30).

Items 3, 5, and 10 are reverse scored (i.e., 3, 2, 1, and 0). The total score is found by adding together the scores for each of the 10 items (Cronbach’s *α*=0.77). The majority of studies utilizing the EPDS use a cutoff score greater than 12 for defining the mothers at risk for depression (Murray and Carothers, 1990) although other cut off scores have been used (Murray and Cox, 1990). Following the indications of the specific literature on depression assessment in the NICU mothers could be considered as at risk for developing postpartum depression when the EPDS were ≥10 (e.g., Teissèdre and Chabrol, 2004; Alkozei et al., 2014).

The PSS:NICU (Miles et al., 1993; Montirosso et al., 2012) was used to assess mothers’ perception of stressors originating from the physical and psychosocial environment in the NICU. The instrument includes three dimensions (subscales): Sights and Sounds of the unit (six items), Infant Behaviour and Appearance (17 items), and Parental Role Alteration (11 items): Items were rated on a Likert-type scale ranging from 1 “not at all stressful” to 5 “extremely stressful.” A total averaged score was computed for the three subscale, and Overall Stress Level has been computed by the average of all the items; the items rated as “not applicable” were coded as 1 (Montirosso et al., 2012). A cutoff score of ≥3 is used to identify high parental stress (Miles et al., 1993; Montirosso et al., 2012) in the total PSS:NICU scores. The reliability of the scale was good both for the subscales (Sights and Sounds Cronbach’s *α*=0.71; Infant Behaviour and Appearance *α*=0.80; Parental Role Alteration *α*=0.86), and the Overall Stress Level (*α*=0.91).

Maternal attachment was assessed using a self-reported questionnaire, the Maternal Postnatal Attachment Scale (MPAS; Condon and Corkindale, 1998). The instrument consists of 18 items. A total score was obtained by the sum of all the answers (Cronbach’s *α*=0.61), and higher score denotes greater attachment.

### Data Analysis

The EPDS, PSS:NICU stress scores and MPAS have been compared in the two groups, Before- and During-COVID, with *t*-tests. In addition, a threshold for at “risk mothers” was applied for EPDS scores equal or greater than 10 and for PSS:NICU scores equal or greater than 3. The numbers of the mothers at risk were then compared in the two groups with Fisher’s exact test instead of chi-square because of the small sample size. Finally, the correlation between EPDS, PSS:NICU stress scores, and MPAS scores was tested using Pearson’s correlations separately for each of the two groups.

All the analyses were conducted with SPSS Statistics 27.

Results were considered as significant at *p*<0.05.

## Results

The two groups were homogeneous and comparable in terms of preterm infant’s neonatal characteristics and for the maternal characteristics (see Table 1), and no significant differences emerged at the *t*-tests comparison between the two groups. No significant differences were found when comparing *t*-tests between the two groups for the SES score [(M=4.90, SD=2.99) for the Before-COVID group and 6.14 (SD 1.75) for the During-COVID group, respectively; *p*=0.17].

### Comparison of EPDS, PSS:NICU, MPAS Scores, and Before- Versus During-COVID

The mean and standard deviations for the EPDS, PSS:NICU subscales, and MPAS are presented in Table 2 for both groups, Before- and During- COVID. No significant results were found in the comparisons between the two groups.

**Table 2:** Means and standard deviations for the study variables in the Before- and During-COVID groups.

	Before-COVID Mean (SD; *N*=20)	During-COVID Mean (SD; *N*=14)	*p*
EPDS	8.45 (1.9)	10.53 (1.8)	0.13
Total PSS:NICU score	3.26 (0.69)	3.34 (0.73)	0.86
PSS: Sights and Sounds	2.91 (0.81)	2.73 (0.75)	0.52
PSS: Infant Behavior and Appearance	2.76 (0.88)	3.05 (0.83)	0.34
PSS: Parental Role Alterations	2.97 (1.0)	2.82 (0.88)	0.64
MPAS	87.16 (5.85)	86.56 (5.15)	0.50

### Mothers “At Risk” in the Before-COVID and During-COVID Periods

Considering the limited samples size (the absence of a significant difference at the *t*-test comparisons of the global scores summed and averaged on these two scales), a non-parametric and more sensible test has been conducted on the number of mothers at risk or not of the two groups, Before and During-COVID. Results indicated non-significant differences for EPDS and PSS (EPDS *p*=0.138; PSS *p*=0.177, Fisher’s Exact tests). However, a trend is present (see Table 3) with a lower percentage of mothers in the “at risk” category for higher stress (43.9%) in the Before-COVID group than in the During-COVID group (57.1%). Similarly, for the EPDS score, the mothers at risk of postpartum depression increase from 45.9 to 68.8%.

**Table 3:** Number and percentage of mothers in the “at risk” categories for postpartum depression higher stress.

	Before-COVID		During-COVID	
**EPDS score**				
Below 10	11	55.00%	5	31.3%
Equal or above 10	9	45.9%	11	68.8%
**Total PSS_Nicu score**				
Below 3	13	65.0%	6	35.0%
Equal or above 3	7	43.9%	8	57.10%

### Association Between EPDS, PSS:NICU, and MPAS Scores

EPDS scores were positively and significantly correlated with total PSS:NICU scores, as well as with two PSS:NICU subscores – Parental Role Alterations, and Infant Behavior and Appearance, but not with Sights and Sounds scores, in the Before-COVID group.

The PSS:NICU Sights and Sounds score is significantly positively correlated with EPDS scores and negatively with the MPAS score only in the in the During-COVID group. PSS:NICU Sights and Sounds score, EPDS, and MPAS scores were independent in the Before-COVID group.

Negative correlations, albeit non-significant, emerged between PSS:NICU and MPAS scores in both groups, while they were negatively correlated in the During-COVID group.

Correlations between variables in the two groups are presented in Table 4.

**Table 4:** Pearson’s correlations for the study variables in the Before- and During-COVID groups.

	EPDS	Total PSS:NICU	PSS: Sights and Sounds	PSS: Infant Behavior and Appearance	PSS: Parental Role Alterations	MPAS
**Before-COVID**						
EPDS score	−	**0.46** ^*^	−0.15	**0.55** ^*^	**0.48** ^**^	
Total PSS:NICU score		−				
PSS: Sights and Sounds		**0.44** ^*^	−			
PSS: Infant Behavior and Appearance		**0.91** ^***^	0.28	−		
PSS: Parental Role Alterations		**0.87** ^***^	0.21	**0.68** ^***^	−	
MPAS score	0.01	−0.14	−0.12	−0.08	−0.19	−
**During-COVID**						
EPDS score	−	**0.65** ^*^	**0.67** ^**^	**0.53** ^*^	**0.70** ^**^	
PSS:NICU score		−				
PSS: Sights and Sounds		**0.78** ^***^	−			
PSS: Infant Behavior and Appearance		**0.96** ^***^	**0.63** ^*^	−		
PSS: Parental Role Alterations		**0.93** ^***^	**0.69** ^**^	**0.86** ^**^		
MPAS score	−0.44	−0.44	**−0.64** ^*^	−0.35	−0.35	−

* *p<0.05*,

** *p<0.01*,

*** *p<0.001*.

*Bold values represent the significant correlations*.

Raw data will be made available by the authors, without undue reservation, to any qualified researcher.

## Discussion

In the present study, two cohorts of mothers, delivering preterm infants of less than 32weeks of gestational age before and during the COVID pandemic, were compared in terms of depression after infant’s birth, and for stress and attachment at hospital discharge. We hypothesized that the COVID-19 pandemic could have a detrimental impact on maternal depression at birth, on maternal stress symptoms during their hospital stay in the NICU, and, consequently, on maternal attachment at hospital discharge.

No significant difference was found in the comparisons between the two groups, but a trend of increased depression, stress, and of decreased attachment scores was found in mothers during the COVID period when compared to the period before the COVID pandemic.

Similarly, analyses of the percentage of mothers classified as “at risk” (Montirosso et al., 2012; Alkozei et al., 2014; data reported in Table 3) showed no significant differences between the two groups. However, an increase in the percentage of mothers in the “at risk” category for depression and for higher-stress is found in the during-COVID period.

In the correlation analyses, maternal depression score was positively and significantly correlated with total PSS:NICU scores, and with two PSS:NICU subscores – Parental Role Alterations, and Infant Behavior and Appearance before the COVID pandemic, confirming previous findings (Alkozei et al., 2014). All these correlations were stronger in the During-COVID group than in the Before-COVID group. Most importantly, it was only during the COVID pandemic that depression scores were significantly associated with the PSS:NICU Sights and Sounds and, interestingly, also with the attachment scores. This result shows that depression scores measured in mothers shortly after the infant’s birth, and stress for the environment at hospital discharge were more strongly associated to difficulties in attachment during this period of global health crisis.

The PSS:NICU Sights and Sounds is a subscale of the PSS:NICU specifically designed to identify stressors caused by the physical environment of the NICU. This environment, with its constant alarms and unpredictable noises, has well-known short- and long-term deleterious effects on the behavior and development of such vulnerable preterm infants (Graven, 2000; Wachman and Lahav, 2011). Findings of the present study suggest that, during the COVID pandemic, the physical environment of the NICU becomes an even more stressful element that is associated with increased depression and decreased levels of attachment. Preventive and protective actions on the physical environment of the infant and family should therefore be adopted in the NICU – especially during the periods of crisis and health emergency in order to prevent a disruption of infants and dyad well-being (Als et al., 2003; van Veenendaal et al., 2021).

The correlation results confirm the robust relation between maternal stress and depression after birth in the NICU (Alkozei et al., 2014), and the correlation is stronger during the COVID pandemic, with impacts on attachment scores.

Mothers of preterm infants are known to be at increased risk of postpartum depression and findings from systematic reviews and meta-analysis support the association between preterm birth and postpartum depression (Miles et al., 1992; Lefkowitz et al., 2010; de Paula Eduardo et al., 2019), therefore, psychological support for mothers during their child’s NICU stay is recommended (Montirosso et al., 2012). While the health problems associated with a pandemic can be highly stressful for all individuals, research suggests that the psychological impact of these traumas may be more severe for some at risk populations (Boyraz and Legros, 2020; Chaix et al., 2020; Stefana et al., 2020).

Maternal attachment representations toward the prematurely born child are fragile (Forcada-Guex et al., 2011) and protective actions, such as infant and family-centered developmental care, can support the complex attachment process in this delicate period of hospitalization in the NICU (Roué et al., 2017). The present results suggest that this support is even more precious in periods of health crisis.

One of the limitations of the present study is the small sample size. The overmentioned non-significant results could be related to the limited sample size due to the inclusion criteria adopted (specifically, mothers of children born very prematurely and before 32weeks of gestation), which did not allow higher numbers of recruitments during the pandemic period.

Moreover, the questionnaires we adopted for evaluating depression give a partial description of the two of the most diffused questionnaires for evaluating maternal depression during the perinatal period are the EPDS and the BDI (Beck Depression Inventory Lefkowitz et al., 2010). However, for future studies, we suggest to adopt a multi-dimensional cluster of measures, including interviews, questionnaires and physiological measures (i.e., cortisol measures), to better assess the type of impact that this stressful life event could have in mothers and fathers in the NICU.

As using maternal depression as a primary construct to characterize all severe and prolonged distress in the NICU setting, the present study does not account for the type of trauma or for minor and more transient forms of “baby blues” and for other important aspects of distress (Greene et al., 2015). We also suggest including measures to evaluate this impact in terms of post-traumatic stress disorder (Anderson and Cacola, 2017).

Another limitation, and a possible explanation for the fact that we did not find significant differences between the groups, is that the period of mothers’ recruitment started 8months after the pandemic was declared, so we were not able to explore the period of initial stress and the differential effects of the different pandemic waves on maternal mental health. It is possible that 8months after the start of the pandemic, mothers may have become slightly adapted to the stress caused by the pandemic, but we were not allowed to carry on any research project during the initial COVID-19 pandemic period.

However, we can also discuss the present results in terms of effects of the NICU policies and protective actions that have been adopted during the pandemic period at the Geneva University Hospital (HUG). By analyzing point by point the potential negative impacts of the COVID pandemic on the NICU policies affecting parent and family access, and patient care reported in the introduction, we can adopt a synoptic perspective and evaluate the NICU protective policies that were adopted at the HUG during the COVID outbreak period.

At HUG, the NICU is organized in single-family rooms, and open access 24/24h and psychological support has been guaranteed for both parents during all the COVID pandemic period. The pediatric intensive care unit and the NICU were the only two units that were exempted from the visitor restriction. Thus, both parents could be present at the same time with no reduction of their usual participation in the infant’s care. Parents used the same precautions as the health personnel each time they acceded the unit and the use of masks inside the room was mandatory only when a member of the medical-nursing team enters the single-family room. When parents were alone with their babies, they could remove the mask, without interrupting the usual intimacy in communication, the skin-to-skin contact or breastfeeding programs. In case one of the parents was tested positively, a quarantine of 10days was imposed, including 48h without symptoms for both parents. The quarantined parents could communicate with their babies *via* a video conferencing system or video call. During the same period, the nurse-medical team communicated with the parents to inform them of their child’s progress. Mothers, who breastfed during the quarantine period, could continue pumping milk at home and could bring it to the hospital through a family member.

Family-centered care practices were maintained during the COVID pandemic at the HUG, and the overmentioned actions, which were routine practices before the COVID period and maintained during the pandemic, could potentially be protective against the negative effects of the pandemic on stress, depression and attachment scores in mothers of hospitalized preterm infants.

## Conclusions

Several protective actions could be adopted in the NICUs during the pandemic period.

In this phase, it becomes imperative to assess parental mental health and to enhance psychosocial support of NICU parents, assuring timely information and finding alternative solutions for parents who cannot visit their infants. Technological devices can be implemented in case of forced separation, in order to maintain the mother’s and father’s visual and vocal contact with their baby (Epstein et al., 2017).

Early family-based interventions, such Early Vocal Contact in the NICU (Arnon et al., 2014; Filippa et al., 2019), can support parent’s sensitive behaviors, increasing their emotional availability, and decreasing stress and anxiety levels during their stay in the NICU. Safely enhancing contact within the dyads or triads, during periods of general isolation from peers and from larger familiar supports, becomes essential (Tscherning et al., 2020). Single family-rooms and individualized newborn care could contain the negative impact of the COVID pandemic (Mahoney et al., 2020). Evidence-based indications on how to safely maintain family-centered developmental care practices in the NICU during the COVID pandemic have been provided (Tscherning et al., 2020; Cena et al., 2021). Future studies are encouraged, in order to evaluate specific protective interventions and policies to be adopted during future possible pandemic experiences or health social crisis.

## Data Availability Statement

The original contributions presented in the study are included in the article/Supplementary Material, and further inquiries can be directed to the corresponding authors.

## Ethics Statement

The studies involving human participants were reviewed and approved by the Swiss Ethical Committee Protocol CCER 2015-00175 (15-295). Written informed consent to participate in this study was provided by the participants’ legal guardian/next of kin.

## Author Contributions

FM conceived the presented idea and wrote the draft. FM, FB-M, LL, and CB-T developed the theoretical framework. FB-M, MC, and JA collected data. MM performed the analyses. All the authors provided critical feedback and contributed to the final version of the manuscript. All authors contributed to the article and approved the submitted version.

## Funding

Fundings from the Swiss National Science Foundation, FNS 320030B_182832, title Building the path to resilience after preterm birth: cortical development and network based interventions in longitudinal cohorts. Additional fundings from Art-Thérapy and PrimEnfance Foundations.

## Conflict of Interest

The authors declare that the research was conducted in the absence of any commercial or financial relationships that could be construed as a potential conflict of interest.

## Publisher’s Note

All claims expressed in this article are solely those of the authors and do not necessarily represent those of their affiliated organizations, or those of the publisher, the editors and the reviewers. Any product that may be evaluated in this article, or claim that may be made by its manufacturer, is not guaranteed or endorsed by the publisher.
